# Risk factors for rebleeding and mortality following prophylactic transarterial embolization for patients with high-risk peptic ulcer bleeding: a single-center retrospective cohort study

**DOI:** 10.1007/s00464-024-10709-x

**Published:** 2024-02-27

**Authors:** Dennis Zetner, Ida Roost Rasmussen, Camilla Palmquist Frykman, Lasse Rehné Jensen, Ruben Juul Jensen, Emma Possfelt-Møller, Mikkel Taudorf, Luit Penninga

**Affiliations:** 1 Department of Radiology, North Zealand Hospital, Copenhagen University Hospital, Hilleroed, Denmark; 2 Department of Surgery, Hvidovre Hospital, Copenhagen University Hospital, Hvidovre, Denmark; 3 Department of Radiology, Gentofte Hospital, Copenhagen University Hospital, Copenhagen, Denmark; 4 Department of Surgery and Transplantation, Rigshospitalet, Copenhagen University Hospital, Copenhagen, Denmark; 5 Department of Radiology, Rigshospitalet, Copenhagen University Hospital, Copenhagen, Denmark; 6 Department of Clinical Medicine, Copenhagen University, Copenhagen, Denmark

**Keywords:** Peptic ulcer bleeding, Embolization, Endovascular, Prophylactic, Rebleeding, Transarterial

## Abstract

**Background:**

To investigate factors associated with risk for rebleeding and 30-day mortality following prophylactic transarterial embolization in patients with high-risk peptic ulcer bleeding.

**Methods:**

We retrospectively reviewed medical records and included all patients who had undergone prophylactic embolization of the gastroduodenal artery at Rigshospitalet, Denmark, following an endoscopy-verified and treated peptic Sulcer bleeding, from 2016 to 2021. Data were collected from electronic health records and imaging from the embolization procedures. Primary outcomes were rebleeding and 30-day mortality. We performed logistical regression analyses for both outcomes with possible risk factors. Risk factors included: active bleeding; visible hemoclips; Rockall-score; anatomical variants; standardized embolization procedure; and number of endoscopies prior to embolization.

**Results:**

We included 176 patients. Rebleeding occurred in 25% following embolization and 30-day mortality was 15%. Not undergoing a standardized embolization procedure increased the odds of both rebleeding (odds ratio 3.029, 95% confidence interval (CI) 1.395–6.579) and 30-day overall mortality by 3.262 (1.252–8.497). More than one endoscopy was associated with increased odds of rebleeding (odds ratio 2.369, 95% CI 1.088–5.158). High Rockall-score increased the odds of 30-day mortality (odds ratio 2.587, 95% CI 1.243–5.386). Active bleeding, visible hemoclips, and anatomical variants did not affect risk of rebleeding or 30-day mortality. Reasons for deviation from standard embolization procedure were anatomical variations, targeted treatment without embolizing the gastroduodenal artery, and technical failure.

**Conclusions:**

Deviation from the standard embolization procedure increased the risk of rebleeding and 30-day mortality, more than one endoscopy prior to embolization was associated with higher odds of rebleeding, and a high Rockall-score increased the risk of 30-day mortality. We suggest that patients with these risk factors are monitored closely following embolization. Early detection of rebleeding may allow for proper and early re-intervention.

**Graphical abstract:**

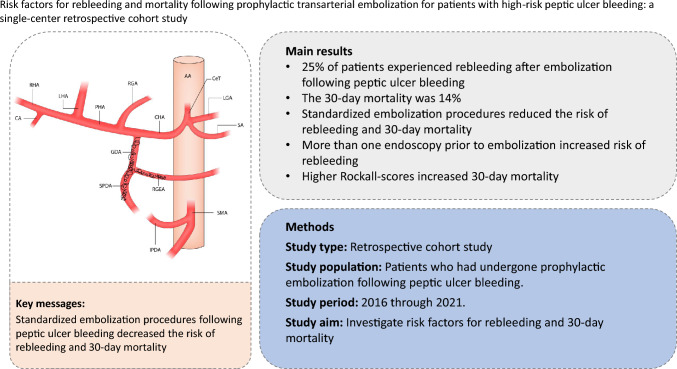

**Supplementary Information:**

The online version contains supplementary material available at 10.1007/s00464-024-10709-x.

Acute upper gastrointestinal bleeding caused by a peptic ulcer is a frequent and life-threatening condition [1]. The gold standard treatment is endoscopic intervention [2]. Rebleeding following treatment for a peptic ulcer bleeding increases mortality significantly [2]. Thus, it is of utmost importance to reduce the risk of rebleeding.

Despite achieving hemostasis endoscopically, 12–25% of patients experience rebleeding, depending on several factors (e.g. ulcer type, patient comorbidities, etc.) [3, 4]. Following successful endoscopic hemostasis, surgeons can consider prophylactic transarterial embolization to reduce the risk of rebleeding. Transarterial embolization decreases both the rate of rebleeding, the need for reintervention, and mortality [5]. Further, transarterial embolization has a high technical success rate, a low complication rate, and does not prolong in-hospital stay significantly [5].

In Denmark, patients do not undergo prophylactic embolization routinely. Thus, it is the surgeon performing the endoscopy, who assesses the need for prophylactic embolization. Among other factors, this evaluation is based on the Rockall score of the patient, and the Forrest classification of the ulcer (Table 1) [6, 7]. Two studies have investigated the effect of prophylactic transarterial embolization following peptic ulcer bleeding in a randomized setup [8, 9]. However, both studies performed the prophylactic arterial embolization after just one endoscopy. In our population, patients were evaluated by surgeons based on the Rockall-score and might have undergone more than one endoscopy [10].

**Table 1:** Rockall Score and Forrest Classification

Rockall score^a^				
Score	0	1	2	3
Age	< 60 years	60–79 years	≥ 80 years	
Shock	No shock (Systolic blood pressure > 100 mmHg and heart rate < 100 bpm)	Tachycardia (Systolic blood pressure > 100 mmHg and heart rate > 100 bpm)	Hypotension (Systolic blood pressure < 100 mmHg)	
Comorbidity			- Ischemic heart disease - Congestive heart failure - Any major comorbidity	- Renal failure - Liver failure - Disseminated malignancy
Diagnosis	Mallory-Weis tear or no lesion observed	Peptic ulcer disease, erosive esophagitis	Malignancy of upper gastrointestinal tract	
Stigmata of recent bleeding	Clean-based ulcer, flat pigmented spot		Blood in upper gastrointestinal tract, clot, visible vessel, bleeding	
Forrest Classification				
Type 1	Active bleeding			
	Ia: Spurting hemorrhage			
	Ib: Oozing hemorrhage			
Type 2	Signs of recent bleeding			
	IIa: Non-bleeding visible vessel			
	IIb: Adherent clot on lesion			
	IIc: Hematin-covered lesion			
Type 3	Lesion without bleeding			
	(flat spot, clean base)			

*bmp* beats per minute

^a^Maximum Rockall-score obtainable is 11

The aim of this study was to retrospectively analyze patients who have undergone prophylactic transarterial embolization at Rigshospitalet, Denmark, following a peptic ulcer bleeding. We aimed to evaluate the risk of rebleeding and 30-day mortality following embolization, and identify risk factors for rebleeding and mortality.

## Materials and methods

The study was a retrospective, single-center cohort study. The study was approved by the local ethics committee of the Capital Region of Denmark (R-22007493). The study was reported according to the Strengthening the Reporting of Observational Studies in Epidemiology (STROBE) Statement: guidelines for reporting observational studies [11].

The study was conducted at Rigshospitalet, Copenhagen, Denmark. Rigshospitalet is a referral center for Zealand, with a population of approximately 2.6 million inhabitants. Patients with peptic ulcer bleeding are typically treated endoscopically at the local and regional hospitals, and referred to Rigshospitalet for embolization. Data collection was performed between September 21 and December 28, 2022. We included all patients who had undergone attempted prophylactic transarterial embolization at Rigshospitalet between January 1, 2016, and December 31, 2021. Patients were followed up until December 28, 2022. For patients to be included into the study, they had to have undergone attempted transarterial embolization following a peptic ulcer bleeding, within the same hospital admission. Patients were excluded if they had incomplete medical records. Patients were identified through a local registry at the Department of Radiology, Rigshospitalet. Data were collected through electronic health records as well as radiological imaging software. Follow-up was also performed through the electronic health records.

**Fig. 1:**
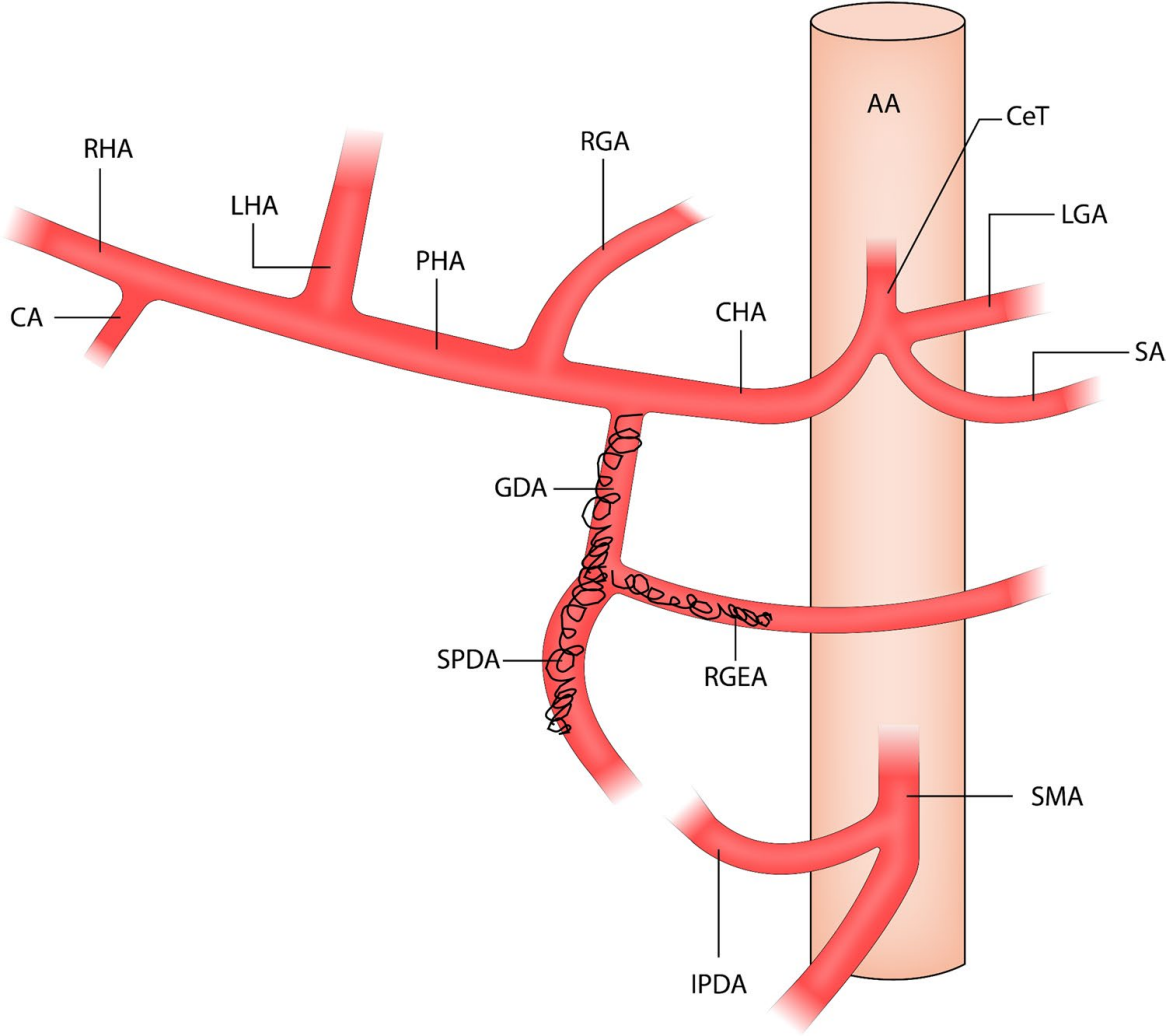
Overview of arteries involved in prophylactic transarterial embolization with embolization of the gastroduodenal artery and side branches. *AA* abdominal aorta, *CeT* celiac trunk, *LGA* left gastric artery, *SA* splenic artery, *CHA* common hepatic artery, *RGA* right gastric artery, *PHA* proper hepatic artery, *LHA* left hepatic artery, *RHA* right hepatic artery, *CA* cystic artery, *GDA* gastroduodenal artery, *SPDA* superior pancreaticoduodenal artery, *RGEA* right gastroepiploic artery, *SMA* superior mesenteric artery, *IPDA* inferior pancreacitoduodenal artery

The following variables were extracted from the patient history in the electronic health record: age, sex, comorbidities, smoking and alcohol history, plasma hemoglobin prior to upper endoscopy, weight, height, use of non-steroidal anti-inflammatory drugs and anti-coagulants, blood pressure and pulse prior to upper endoscopy (pulse < 100, systolic blood pressure > 100; pulse > 100, systolic blood pressure > 100; systolic blood pressure < 100), American Society of Anaesthesiologists score [12], time of last upper endoscopy prior to embolization, placement of ulcer, size of ulcer, endoscopic method of bleeding control, if bleed control was achieved (yes/no), Forrest classification [7], diagnosis as per the Rockall-score [6], Helicobacter pylori status, number of upper endoscopies prior to embolization, rebleed after embolization, date of rebleed, need for reintervention, type of reintervention, death at time of follow-up, date of death, cause of death, discharge date from hospital, readmission related to bleeding ulcer, and date of readmission. The following variables were extracted from the digital subtraction angiography (DSA) by a trained interventional radiologist: time of embolization, active bleeding on digital subtraction angiography, visible hemoclips during digital subtraction angiography, type of embolization material, standard embolization (yes/no), any anatomic variants on digital subtraction angiography, visualization of cystic artery (yes/no), embolization of cystic artery, non-target embolization (yes/no). We defined a standard embolization as coiling of the gastroduodenal artery and side branches, as well as any actively bleeding sites (Fig. 1), as this is the standard embolization technique employed at our institution. Consequently, non-standard embolization includes all embolizations not following the above, e.g., attempted embolizations with technical failure, anatomical variants making a standard approach impossible, different embolization materials. All data were entered into a Research Electronic Data Capture (REDCap) database.

Normally distributed data were reported as mean (SD) and non-normally distributed data were reported as median (range). The Rockall-score was grouped (≤ 3, 4–5, ≥ 6) based on the Danish Society of Gastroenterology and Hepatology guidelines [10]. Both primary outcomes (rebleeding and 30-day overall mortality) were dichotomous outcomes. To investigate the association between rebleeding and 30-day overall mortality, we performed a *χ*^2^-test. If there was an association, we then calculated the odds ratio to quantify the association. Finally, we performed a binomial logistical regression, evaluating the effect of several variables on the likelihood of rebleeding and 30-day mortality. The variables included in the model were: active bleeding during transarterial angiography, visible hemoclips following endoscopy during transarterial angiography, Rockall score (grouped), normal anatomy, standard embolization procedure, and number of endoscopies performed prior to embolization (grouped into either one endoscopy or more than one endoscopies). Results with *p* < 0.05 were considered statistically significant.

## Results

**Fig. 2:**
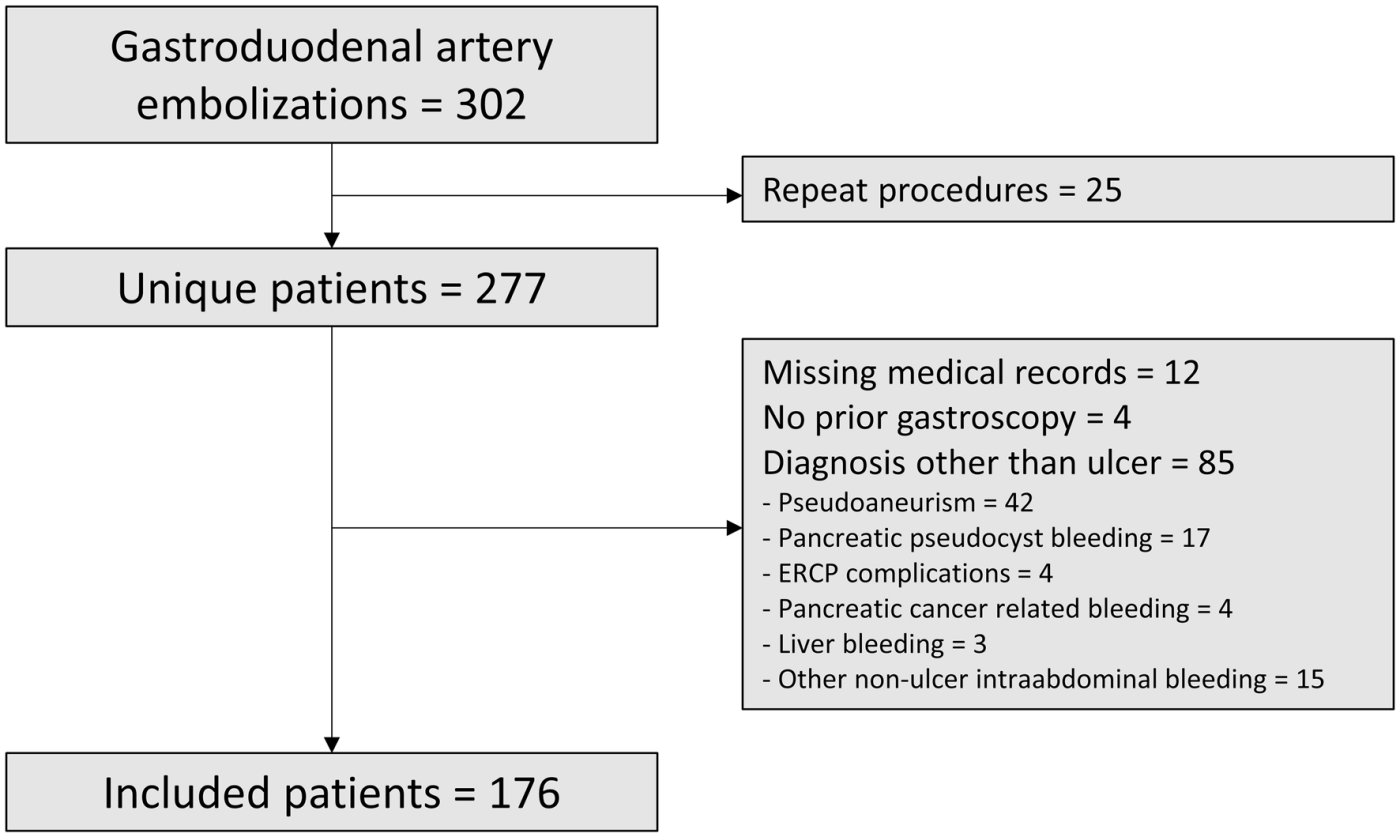
Flowchart of cohort selection. *ERCP* endoscopic retrograde cholangiopancreatography

We identified a total of 302 transarterial embolizations performed at Rigshospitalet from 2016 to 2021. Of these, 277 were unique patients. We excluded 101 patients for the following reasons: 12 were missing medical records, 85 had other diagnoses than peptic ulcer bleeding, and 4 had not had an upper endoscopy prior to transarterial embolization. In total, 176 patients were included in the study, see Fig. 2. All embolizations were performed by one of seven experienced interventional radiologists at Rigshospitalet. For descriptive and outcome data, see Table 2.

**Table 2:** Characteristics and outcomes of patients included

Outcome	Result	Missing data, *n (%)*
Total included, *n*	176	
Age, median (range)	73 (41–103)	0
Sex (male), *n* (%)	113 (64)	0
Comorbidities, *n* (%)		
Diabetes	40 (23)	0
COLD	35 (20)	0
Heart disease	72 (41)	0
Liver disease	17 (10)	0
Liver failure	10 (56)	
Kidney disease	37 (21)	0
Kidney failure	26 (72)	
Upper gastrointestinal cancer	7 (4)	0
Disseminated cancer	19 (11)	0
Smoking		2 (1)
Current	66 (38)	
Previous	58 (33)	
Never	50 (29)	
Excessive alcohol consumption, *n* (%)^a^	44 (25)	2 (1)
Medications *n* (%)		
Aspirin	35 (20)	0
Anticoagulantia	64 (36)	0
NSAIDs	24 (14)	1 (1)
Clinical data		
Hemodynamic shock, *n* (%)^b^	82 (47)	1 (1)
Hemoglobin (mmol/L), mean (SD)^b^	4.64 (0.98)	0
ASA-score, median (range)^b^	3 (1–4)	19 (11)
Location of ulcer		0
Duodenal ulcer, *n* (%)	168 (96)	
Forrest classification, *n* (%)		7 (4)
1a	51 (29)	
1b	58 (33)	
2a	21 (12)	
2b	24 (14)	
2c	8 (5)	
3	7 (4)	
Rockall score, median (range)	7 (2–10)	1
≤ 3, *n* (%)	6 (3)	
4–5, *n* (%)	30 (17)	
> 5, *n* (%)	140 (80)	
Number of endoscopies prior to coiling, n (%)		0
1	79 (45)	
2	65 (37)	
3	23 (13)	
4	9 (5)	
Outcomes		
Hours from endoscopy to embolization, mean (IQR)	23 (9–47)	0
Rebleeding, *n* (%)	44 (25)	0
Reintervention, *n* (%)	39 (91)	1 (2)
Upper endoscopy, *n* (%)	29 (74)	0
Embolization, *n* (%)	17 (44)	0
Surgery, *n* (%)	11 (28)	0
Readmission within 30 days, *n* (%)	4 (2)	0
In-house mortality (bleeding related), *n* (%)^c^	15 (9)	0
In-house mortality (overall), *n* (%)^c^	27 (15)	0
30-day mortality (bleeding related), *n* (%)	14 (8)	0
30-day mortality (overall), *n* (%)	26 (15)	0
Follow-up, months, *median* (range)	19 (0–78)	0
Hospital stay, days, *median* (range)^c^	9 (1–187)	0
Complications due to ischemia, *n* (%)	0 (0)	0
Embolization		
Active bleeding during arteriography, *n* (%)	33 (19)	0
Visible hemoclips left from endoscopy, *n* (%)	114 (65)	0
Standard embolization (GDA + branches), *n* (%)	154 (88)	0
Normal anatomy, *n* (%)	111 (63)	0
Visualization of cystic artery, *n* (%)	40 (23)	0
Coiling of cystic artery, *n* (%)	3 (8)	0
Non-target embolization, *n* (%)	9 (5)	0
Embolization material		
Coils, *n* (%)	172 (98)	0
Glue, *n* (%)	6 (3)	0
Spongostan, *n* (%)	1 (1)	0
Plugs, *n* (%)	7 (4)	0
Other, *n* (%)	4 (2)	0
Only arteriography	1	0
Onyx	1	0
Particles	1	0
Macrocoils and vascular plugs	1	0

*COLD* chronic obstructive lung disease, *NSAID* non-steroidal anti-inflammatory drugs, *SD* standard deviation, *ASA* American Society of Anaesthesiologists, *IQR* inter-quartile range, *GDA* gastroduodenal artery

^a^Defined as over 14 units of alcohol per week

^b^Evaluated just before the last endoscopy prior to embolization

^c^In-house was defined as during the same hospital admission, including transfers between Rigshospitalet, and the referring hospital

Of the 26 patients who died within 30 days of embolization, 15 (58%) had experienced a rebleeding following embolization. The *χ*^2^-test of independence showed an increased likelihood of 30-day mortality in patients who had suffered a rebleeding following transarterial embolization (*p* < 0.001), and the odds ratio of 30-day mortality in patients who had suffered a rebleeding following transarterial embolization was 5.6897 (95% confidence interval 2.3665–13.6795).

None of the included patients had significant complications directly related to the transarterial embolization, i.e., none suffered from ischemia or perforation, and no complications arose in the patients who had non-target embolization or coiling of the cystic artery.

The rebleeding binomial logistic regression model was statistically significant, *χ*^2^ (6) = 19.207, *p* = 0.004. The model explained 15.3% (Nagelkerke *R*^2^) of the variance in rebleeding and correctly classified 76.7% of cases. Sensitivity was 18.2%, specificity was 96.2%, positive predictive value was 61.5% and negative predictive value was 77.9%. Of the six predictor variables (active bleeding, visible hemoclips, Rockall score, normal anatomy, standard embolization procedure, and number of endoscopies prior to embolization) two were statistically significant, standard embolization procedure and number of endoscopies prior to embolization, see Table 3. Patients who did not undergo a standard embolization procedure had 3.298 (1.484–7.329) higher odds (95% confidence interval) of rebleeding compared with patients who underwent a standard embolization procedure. Patients who had more than one endoscopy prior to embolization had 2.369 (1.088–5.158) higher odds (95% confidence interval) of rebleeding compared with patients who only had one endoscopy.

**Table 3:** Logistic regression predicting likelyhood of rebleeding based on active bleeding, visible haemoclips, Rockall score, normal anatomy and standard embolization procedure

	*B*	SE	Wald	*p*	Odds Ratio	95% CI for Odds Ratio	
						Lower	Upper
Active bleeding	− 0.550	0.446	1.519	0.218	0.577	0.241	1.383
Visible haemoclips	0.507	0.389	1.695	0.193	1.659	0.774	3.557
Rockall score	0.179	0.375	0.228	0.633	1.196	0.574	2.492
Normal anatomy	0.268	0.386	0.483	0.487	1.308	0.614	3.082
Standard embolization procedure	1.193	0.407	8.583	**0.003**	3.298	1.484	6.579
> 1 endoscopy before embolization	0.862	0.397	4.720	**0.030**	2.369	1.088	5.158
Constant	− 4.335	1.622	7.142	0.008	0.013		

*B* unstandardized regression weight, *SE* standard error, *Wald* Wald test, *CI* confidence interval

The bold values are highlighted because they show statistically significant *p*-values

The 30-day overall mortality binomial logistic regression model was not statistically significant, *χ*^2^ (6) = 12.082, *p* = 0.06. However, the Hosmer and Lemeshow goodness of fit test was not significant (*p* = 0.379), indicating that the model is not a poor fit, for predicting categorical outcomes. The model explained 11.7% (Nagelkerke *R*^2^) of the variance in 30-day overall mortality and correctly classified 85.8% of cases. Sensitivity was 3.8%, specificity was 100%, positive predictive value was 100% and negative predictive value was 85.7%. Of the six predictor variables (active bleeding, visible hemoclips, Rockall score, normal anatomy, standard embolization procedure, and number of endoscopies prior to embolization) two were statistically significant, Rockall score and standard embolization procedure, see Table 4. Patients who underwent a non-standard embolization procedure had 3.340 (1.273–8.765) higher odds (95% confidence interval) of 30-day mortality compared with patients who underwent a standard embolization procedure. Further, a higher Rockall score was associated with an increased likelihood of 30-day mortality.

**Table 4:** Logistic regression predicting likelyhood of 30-day mortality based on active bleeding, visible haemoclips, Rockall score, normal anatomy and standard embolization procedure

	*B*	SE	Wald	*P*	Odds Ratio	95% CI for Odds Ratio	
						Lower	Upper
Active bleeding	0.320	0.591	0.293	0.588	1.378	0.432	4.391
Visible haemoclips	0.259	0.465	0.310	0.578	1.295	0.521	3.220
Rockall score	0.949	0.377	6.328	**0.012**	2.584	1.233	5.414
Normal anatomy	0.080	0.477	0.028	0.867	1.083	0.425	2.757
Standard embolization procedure	1.206	0.492	6.001	**0.014**	3.340	1.273	8.765
> 1 endoscopy before embolization	0.388	0.461	0.711	0.399	1.475	0.598	3.636
Constant	− 6.240	2.037	9.384	0.002	0.002		

*B* unstandardized regression weight, *SE* standard error, *Wald* Wald test, *CI* confidence interval

The bold values are highlighted because they show statistically significant *p*-values

Finally, we performed a qualitative analysis of embolizations defined as non-standard. In Table 5, reasons for them being defined as non-standard are listed, and the procedures were divided according to if the patient experienced rebleeding following the non-standard embolization or not.

**Table 5:** Reasons for embolizations to be considered non-standard, divided into patients who experienced rebleeding following embolization

	Rebleed	No rebleed
Anatomical variant	4	1
Technical failure^a^	3	0
Targetted treatment without coiling of GDA	2	10
Other embolization material than coils	0	2
Total	9	13

*GDA* gastroduodenal artery

^a^Technical failure was e.g. not embolizing near the hemoclips, or insufficient packing of coils

## Discussion

This retrospective cohort study included 178 patients who underwent prophylactic transarterial embolization following endoscopic treatment for peptic ulcer bleeding. Following transarterial embolization, 25% of patients suffered from rebleeding and overall 30-day mortality was 14%. The risk of 30-day mortality was significantly increased in patients who suffered from rebleeding. Not undergoing a standard treatment, embolization of the gastroduodenal artery and side branches as well as actively bleeding sites (if any), increased the odds of rebleeding by 3.298, and the odds of dying within 30 days of the procedure by 3.340. Having undergone more than one endoscopy prior to embolization increased the odds of rebleeding by 2.369, and a high Rockall-score at the time of the last endoscopy prior to transarterial embolization was associated with a high likelihood of overall 30-day mortality.

A strength of this study is the near complete follow-up of all patients due to electronic health records and the unique Danish patient identification number system. Furthermore, the authors had access to both surgical reports and digital subtraction angiographies, so procedures could be reevaluated. This study was limited by the nature of a retrospective design, by having no control group, and possible selection bias in the patients who were lost to follow-up due to incomplete medical records.

To our knowledge, there is no definitive consensus on which vessels to embolize, and we found no firm guidelines dictating if the gastroduodenal artery always should be embolized, even when a visible actively bleeding vessel is identified during the digital subtraction angiography [5, 13, 14].

Compared with previous studies, we had a high rate of rebleeding, similar mortality rate, and similar adverse event rate [5, 8, 9, 14, 15]. Most patients in our study had undergone more than one endoscopy prior to embolization, which was not the case in most comparable studies. The Rockall scores of our patients were comparable with another retrospective study [15], but our rate of rebleeding was higher. Compared with the randomized clinical trial, also performed in Denmark, most patients in the current study had undergone more than one endoscopy, their clinical condition was worse, and they had more comorbidities [8]. Overall, we attribute the higher rate of rebleeding in our cohort to the worse overall clinical condition of our patients, possibly due to delay of embolization as patients had undergone more endoscopies prior to embolization. As in other studies, we found no adverse events specifically related to the transarterial embolization procedure in our study.

In general, characeristics of patients, who undergo prophylactic transarterial embolization following peptic ulcer bleeding, might vary extensively between different countries. This to a high extent depends on the accessibility of endovascular treatment. Naturally, this limits the external validity of our study.

In conclusion, our study in patients with high-risk peptic ulcer showed a high risk of rebleeding (25%) and mortality (15%) following prophylactic transarterial embolization, with higher likelihood of mortality in patients with rebleeding. We found that patients who had undergone a non-standard prophylactic transarterial embolization had a higher risk of rebleeding and 30-day mortality, patients who had undergone more than one endoscopy prior to embolization had higher odds of rebleeding, and patients with a high Rockall-score had a higher risk of 30-day mortality. However, comparison between our study population and the populations of previous trials was difficult, warranting further research to identify patients who benefit from prophylactic transarterial embolization, and to identify patients at high risk of rebleeding and mortality.

### Supplementary Information

Below is the link to the electronic supplementary material.

Supplementary file1 (DOCX 18 kb)

Supplementary file2 (DOCX 35 kb)
