# Supplementary material for: Risk factors for rebleeding and mortality following prophylactic transarterial embolization for patients with high-risk peptic ulcer bleeding: a single-center retrospective cohort study
Source: Surg Endosc. 2024 Feb 27;38(4):2010–8. doi: 10.1007/s00464-024-10709-x (PMC10978705; doi:10.1007/s00464-024-10709-x)
Supplement: Supplementary file 1 — Supplementary file1 (DOCX 18 kb) [file 464_2024_10709_MOESM1_ESM.docx]

**Protocol**

**Titel:** Profylactic arterial embolization for bleeding peptic ulcers

**Group:** Ida Roost Rasmussen, Mikkel Taudorf, Luit Penninga

**Background**Upper gastrointestinal bleeding is a common emergency in gastroenterology. In Denmark we have around 1300 patients a year and a mortality of around 10%pr year, which has not changed significantly in many years (Christensen; Ahsberg). The most common cause for upper gastrointestinal bleeding is a peptic ulcer in the ventricle or the duodenum. The aetiology of peptic ulcer is complex, but Helicobacter pylori infection and NSAID use are the most common causes. The source of the bleeding is often from arteries in the submucosa or tunica muscularis, but there can be erosions of arteries like the gastroduodenal artery, the right gastric artery or the left gastric artery.

Patients usually present with hematemesis, melaena or both, and sometimes hemodynamic instability. The initial treatment and assessment of these patients should be by the ABCDE-principles. The top priority is to correct fluid losses and to restore haemodynamic stability. The bleeding episode stops spontaneously in most of them, but there is a high risk of rebleeding. Early endoscopy within 24 hours is recommended. After endoscopic treatment, up to 31% of the patients experience rebleeding. Transarterial embolization(TAE) has been introduced as a part of the treatment in the past two decades. It is mostly used to stop the bleeding when conservative treatment and endoscopic therapy have failed. But because of the low risk of complications from TAE, prophylactic TAE after successful endoscopic therapy has been introduced.

In this retrospective study we will examine the effects of prophylactic embolization. We will look into the rates of rebleeding, complications, morbidity and mortality.

**Purpose**Upper GI-bleeding is a common surgical emergency. In spite of the use of endoscopic treatment and proton pump inhibitor (PPI) treatment, the mortality rate worldwide is still around 5-10%. One of the most severe complications to upper GI-bleeding is re-bleeding, which leads to a four- to five-fold increase in mortality rate. If prophylactic TAE can reduce the rate of re-bleeding it can affect a lot of patients and potentially lower the economical burden in the health care budget.

**Methods**We will conducta retrospective study of all patients who underwent prophylactic embolization of a bleeding peptic ulcer in the period from January, 1^st^ 2016 toJanuary 1^st^ 2021 at Rigshospitalet. The patients will be identified by searching interventionscodes for the procedure as applied by the interventional radiologist. Selection of the patients will be done by applying inclusion- and exclusionscriteria as stated below. Futher data will be extracted by reading electronic health records (EPIC/Sundhedsplatformen) as well as imaging software. Data will be analysed applying SPSS statistics for Windows. Complications will be registered using the Clavien-Dindo classification system.

We will describe patient characteristics including, sex, age, comorbidity, use of NSAID, h. pylori infection, location of ulcera (Gatric vs duodenum), type of ulcera (Forrest classification), Rockall score, type of intervention (adrenalin injection, contact hemostasis, metal clips), …………

**Patients**Inclusion criteria

- All Patients who underwent prophylactic arterial embolization for bleeding peptic ulcer after successful endoscopic treatment in the period from 01.01.2017-01.01.2022 at Rigshospitalet

**Outcome**

**Primary**

- **Mortality**
- **Rebleeding**

**Secondary**

- **Risk of reintervention (endoscopy, surgery embolization),**
- **Adverse events (Clavien.Dindo Classification)**
- **Length of hospital stay**

Christensen S, Riis A, Nørgaard M, Sørensen HT, Thomsen RW. Short-term mortality after perforated or bleeding peptic ulcer among elderly patients: a population-based cohort study. *BMC Geriatr*. 2007;7:8. Published 2007 Apr 17. doi:10.1186/1471-2318-7-8

Ahsberg K, Ye W, Lu Y, Zheng Z, Staël von Holstein C. Hospitalisation of and mortality from bleeding peptic ulcer in Sweden: a nationwide time-trend analysis. Aliment Pharmacol Ther. 2011 Mar;33(5):578-84. doi: 10.1111/j.1365-2036.2010.04562.x. Epub 2011 Jan 6. PMID: 21210831.
